# Synergy of Au–Pt for Enhancing Ethylene Photodegradation Performance of Flower-like TiO_2_

**DOI:** 10.3390/nano12183221

**Published:** 2022-09-16

**Authors:** Wanzhen Meng, Yunrui Zhao, Dujuan Dai, Qianqian Zhang, Zeyan Wang, Yuanyuan Liu, Zhaoke Zheng, Hefeng Cheng, Ying Dai, Baibiao Huang, Peng Wang

**Affiliations:** 1 State Key Laboratory of Crystal Materials, Shandong University, Jinan 250100, China; 2 School of Physics, Shandong University, Jinan 250100, China

**Keywords:** surface plasmon resonance, Au–Pt nanoparticles, synergy, non-polar small molecules, photodegradation

## Abstract

Efficient and low-cost degradation of ethylene has always been a difficult problem in the storage and transportation of fruits and vegetables. Although photocatalysis is considered to be a feasible and efficient solution for ethylene degradation, the low degradation ability of conventional catalysts for small non-polar molecules limits its application. TiO_2_ has the advantage of tunable microstructure, but it also has the defects of wide band gap and low utilization of sunlight. The surface plasmon resonance (SPR) effect of noble metals can effectively improve the visible light absorption range of catalysts, and the synergy of noble metals further enhances the photocatalytic ability. Herein, we developed a series of AuPt catalysts through the photo-deposition method. Benefited from the SPR effect and the synergy of Au and Pt, the efficiency of AuPt–TiO_2_ was 19.9, 4.64 and 2.42 times that of TiO_2_, Au–TiO_2_ and Pt–TiO_2_, and the photocatalytic degradation ability of AuPt–TiO_2_ was maintained in five cyclic stability tests. Meanwhile, the transient photocurrent spectra and PL spectra proved that the light absorption capacity and carrier separation efficiency of AuPt–TiO_2_ were enhanced. This work provides a new direction for enhancing non-polar small-molecule photodegradation of semiconductors.

## 1. Introduction

Ethylene, as an endogenous plant hormone, has both positive and negative effects on mature crops. Although ethylene acts positively as a ripening agent, it promotes spoilage of crops and increases transportation and storage costs [1,2,3]. On the basis of refrigeration, controlling the storage and transportation atmospheres of fruits and vegetables is the main method to inhibit the production of ethylene during the storage and transportation of fruits and vegetables. However, refrigeration and specific atmospheres lead to higher costs during storage and transportation. Herein, photocatalytic ethylene oxidation is considered as an efficient and environmentally friendly way to maintain crop quality and reduce transportation and storage costs [4,5,6,7,8]. As a non-polar molecule, ethylene is difficult to be fully mineralized to CO_2_ during the photodegradation process [9,10]. As above, it is necessary to design photocatalysts with strong activation ability for photocatalytic ethylene oxidation [11,12,13,14].

Due to the advantages of low cost, strong ultraviolet light absorption and high stability, TiO_2_ is the most widely studied photocatalyst in the area of hydrogen generation, environmental purification and CO_2_ reduction [15,16,17,18,19,20]. Due to the low quantum conversion and rapid recombination of photogenerated electron–hole pairs and weak visible light absorption, the photocatalytic activities of bare TiO_2_ are limited [21,22,23,24,25]. Various strategies have been developed to promote the photocatalytic efficiency of TiO_2_, such as constructing heterojunctions, doping and supporting cocatalysts. Specifically, the localized surface plasmon resonance (SPR) phenomenon of noble metals has been employed in improving the photocatalytic activity of oxides. For instance, Ag–ZnO with a porous microsphere structure was synthesized in our previous work, and showed ideal activity and good stability as a photodegradation catalyst of methane and ethylene [26]. It has been proven that Ag NPs can boost the separation of photogenerated carriers, accelerate the oxygen reduction and promote the complete mineralization of gas molecules. However, Ag is easily oxidized, limiting its catalytic ability. Moreover, Au has the advantages of strong SPR response and high stability; additionally, Pt is one of the widely used catalysts in the field of photocatalysis due to its small work function and excellent performance. In comparison with sole metals, loading bimetallic noble-metal alloys is more conducive to the improvement of catalyst performance due to their synergy [27,28,29,30]. For example, compared with Au–ZnO and Ag–ZnO, AuAg–ZnO showed better activity and stability for photocatalytic ethylene oxidation. The outstanding activity of ethylene photodegradation was attributed to the synergy of plasmonic AuAg bimetallic alloy nanoparticles [31]. Therefore, the photocatalytic activity of TiO_2_ could be improved by loading bimetallic alloys, which could enhance the visible light absorption due to the SPR effect.

Herein, we synthesized a series of AuPt–TiO_2_ and adjusted the ratio of Au/Pt to obtain optimal photodegradation performance. Due to the synergistic effect of Au and Pt, the visible light absorption capacity and the carrier separation efficiency are improved, and the flower-like microsphere structure of TiO_2_ increases the specific surface area of gas adsorption, which causes AuPt–TiO_2_ to have a remarkably outstanding ethylene photodegradation performance. The performance of the obtained AuPt–TiO_2_ in ethylene photodegradation is 19.9, 4.64 and 2.42 times higher than that of TiO_2_ microspheres, Au–TiO_2_ and Pt–TiO_2_, respectively, and possesses better outstanding ethylene degradation properties. Meanwhile, AuPt–TiO_2_ also showed excellent cycle stability, indicating that AuPt–TiO_2_ is an ideal ethylene photodegradation catalyst.

## 2. Experimental Section

### 2.1. Chemicals

Acetic acid (HAc), tetrabutyl titanate (TBT), tetrachloroauric acid (HAuCl_4_·4H_2_O) and chloroplatinic acid (H_2_PtCl_6_·6H_2_O) were supplied from Sinopharm Chemical Reagent Co., Ltd. (Shanghai, China). Methanol and ethanol were obtained from Aladdin Reagent Corporation (China). All chemicals were of analytical grade and did not require further purification before use.

### 2.2. Synthesis of TiO_2_ Microspheres (TiO_2_ MSs)

The TiO_2_ microspheres were synthesized through a previous hydrothermal reaction with acetic acid (HAc) and tetrabutyl titanate (TBT) [32]. Primarily, 2 mL TBT was tardily added into 60 mL HAc, and stirred vigorously at room temperature for 40 min to form a homogeneous solution. Then, the mixture was placed into a stainless-steel autoclave lined with 100 mL Teflon and heated for 12 h at 150 °C. When cooling to room temperature, the mixture was washed several times after suction filtration with ethanol to obtain a white powder and dried at 70 °C for 6 h in a vacuum oven. Then, the powder was heated at 500 °C for 2 h to obtain TiO_2_ microspheres.

### 2.3. Synthesis of Au or Pt NPs Loaded on TiO_2_ Microspheres

The deposition of single Au NPs on TiO_2_ microspheres was prepared by the process of photo-reduction. Typically, 100 mg TiO_2_ was dissolved in a blend of 20 mL methanol and 80 mL water with the help of ultrasound, stirring for 30 min. Different amounts of HAuCl_4_ (5 mg/mL) solution were dripped into the mixture under dark conditions and stirred vigorously for 20 min. After that, the solution was irradiated at 300 W under a xenon lamp for 30 min. Finally, the mixed solution was filtered, washed with water and ethanol, dried in a vacuum drying chamber at 70 °C for 12 h and calcined for 1 h at 400 °C. By adjusting the volume of HAuCl_4_ solution added, Au–TiO_2_ composites with various mass ratios of Au precursor and TiO_2_ were obtained, named Au_X_–TiO_2_ (X = 0.3, 0.5, 0.8, 1.0). The Au NPs exhibiting superior photocatalytic activity at 0.5 wt% load were selected for the next experiment. The photo-reduction method was also used to support single Pt NPs on TiO_2_ microspheres, but the difference was that a H_2_PtCl_6_ (0.0772 mol/L) solution was used instead.

### 2.4. Synthesis of Au–Pt NPs Loaded on TiO_2_ Microspheres

The process involving Au–Pt bimetallic NPs supported on TiO_2_ microspheres was carried out using a co-photo-reduction method. Similar to the above synthesis steps, different amounts of H_2_PtCl_6_ solution and a fixed amount of HAuCl_4_ solution were simultaneously added to the mixed solution under dark conditions, illuminated, washed, dried and calcined to obtain AuPt–TiO_2_ with different mass ratios of AuPt–TiO_2_ composite material, represented by Au_0.5_Pt_x_–TiO_2_ (x = 0.5, 0.8, …, 1.8). In addition, the bimetallic composite exhibited the most excellent photocatalytic activity under the theoretical loadings of Au = 0.5 wt% and Pt = 1.5 wt% and was named AuPt–TiO_2_. Meanwhile, in order to compare the subsequent photocatalytic performance, single-metal-supported catalysts with the same total loadings were prepared and named Au_2_–TiO_2_ and Pt_2_–TiO_2_.

### 2.5. Characterizations

The microstructures of above-mentioned catalysts were investigated by scanning electron microscopy (SEM) images acquired with a Hitachi S-4800 microscope and transmission electron microscopy acquired with an FEI TalosF200x. The elemental composition of the catalysts was indicated by energy-dispersive X-ray analysis (EDX) data, which were obtained on a Horiba system with a Hitachi S-4800 microscope. Powder X-ray diffraction (XRD) data were obtained with a SmartLab 9 KW in situ X-ray diffractometer for studying the phase and composition of the catalysts. X-ray photoelectron spectroscopy (XPS) performed with a Thermo Scientific ESCALAB 250Xi was used to investigate the surface composition of the obtained samples. To measure UV–vis diffuse reflectance spectra (DRS) for exploring the optical absorption, a Shimadzu UV-2600 spectrophotometer equipped with an integrating sphere was used, with 100% BaSO_4_ as a reflectance standard. The Brunauer–Emmett–Teller (BET) surface area of the catalysts was determined by nitrogen adsorption–desorption isotherms of the samples, which were characterized on a Kubo-X1000 apparatus. The photoluminescence (PL) spectra of catalysts were recorded on an Edinburgh FLS1000 with excitation wavelength at 375 nm.

### 2.6. Photocatalytic Experiments

The activity of ethylene photodegradation was measured by irradiating ethylene gas with light from a 300 W Xe lamp in a 400 mL cylindrical quartz reaction vessel cooled by 15 °C circulating water. First, 50 mg of sample was evenly distributed in the reactor. Secondly, under the conditions of darkness, airtight container and stirring, the reactor was injected with 2 mL (5000 ppm) of ethylene gas. During the dark reaction process, 0.3 mL of gas was extracted from the reactor and injected into a gas chromatograph (Shimadzu GC-2014C) to measure the gas concentration, and the gas was taken three times to obtain the average value of the gas concentration as the initial concentration of ethylene C_0_. Third, the reactor was irradiated by a 300 W Xe lamp on the top of the quartz cover and 0.3 mL of gas was withdrawn from the reactor at regular time intervals and tested by a gas chromatograph to obtain the specific concentration C of ethylene at this time; C/C_0_ expressed the photodegradation percentage of ethylene.

## 3. Results and Discussion

**Figure 1:**
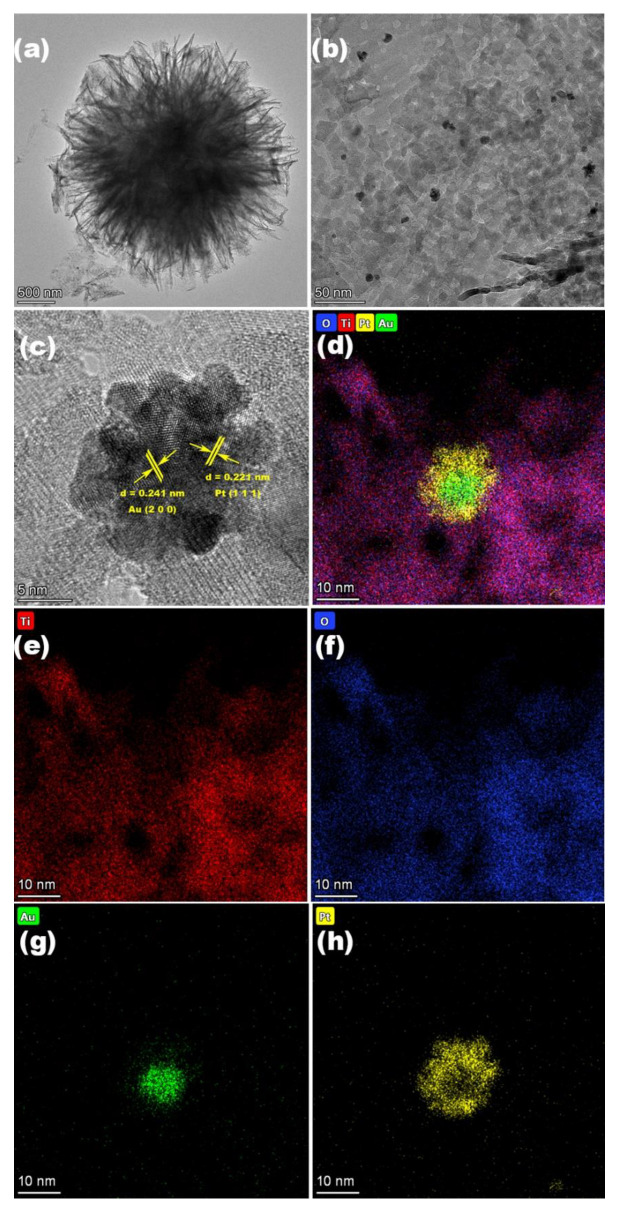
(**a**,**b**) TEM, (**c**) HRTEM images and (**d**–**h**) elemental mapping of AuPt–TiO_2_.

Figure 1a shows that the AuPt–TiO_2_ maintains a microsphere structure consisting of nanosheets, which is consistent with the TiO_2_ MSs (Figure S1). Similarly, the SEM images of Au–TiO_2_ and Pt–TiO_2_ (Figure S2b,c) also display the microsphere structure, revealing that the loading of noble metals has no significant impact on the morphology of TiO_2_. It can be observed from Figure 1b that the Au–Pt nanoparticles are evenly distributed on the nanosheets of TiO_2_ MSs. Furthermore, in the high-resolution TEM (HRTEM) image (Figure 1c), the lattice spacing of 0.352 nm can be attributed to the (101) face of anatase TiO_2_, and the lattice spacing of 0.241 nm and 0.221 nm confirm the existence of Au and Pt. The EDX spectrum (Figure S3a) reveals that the elemental content of Au and Pt is close to the theoretical ratio. The elemental mappings in Figure 1d–f reveal the uniform distribution of Ti, O, Au and Pt. In addition, as shown in Figure S3b,c, the EDS line scan is used to explore the composition of the Au–Pt nanoparticles on the surface of TiO_2_ MSs, which shows that the Pt coated the Au surface to form nanoparticles.

**Figure 2:**
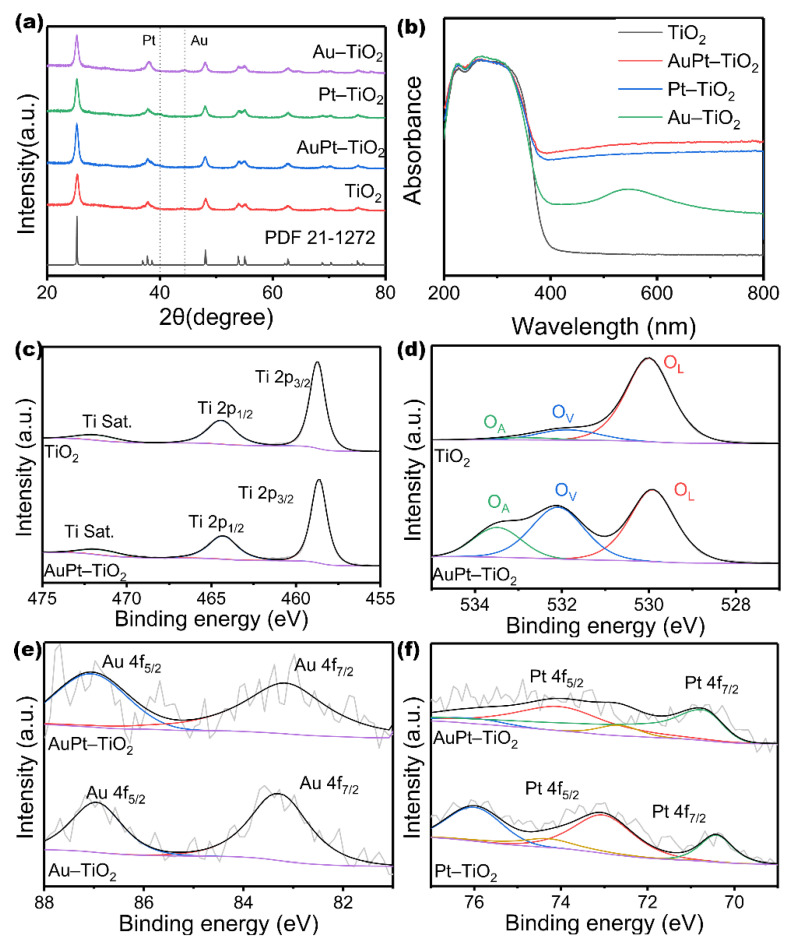
(**a**) XRD patterns and (**b**) UV–vis spectra of TiO_2_ MSs, AuPtTiO_2_, Pt–TiO_2_ and Au–TiO_2_. (**c**) Ti 2p and (**d**) O 1s of TiO_2_ MSs and AuPt–TiO_2_, (**e**) Au 4f of AuPt–TiO_2_ and Au–TiO_2_ and (**f**) Pt 4f XPS spectra of AuPt–TiO_2_ and Pt–TiO_2_.

As shown in Figure 2a, the elemental composition and crystalline phase of catalysts are analyzed by XRD patterns. According to PDF 21-1272, the anatase TiO_2_ is shown to be present in all catalysts. Excluding the characteristic peaks of metals, the diffraction peaks of the modified catalysts are almost similar to those of pure TiO_2_. There is a weak peak at 39.7° shown in the XRD patterns of Pt–TiO_2_ and AuPt–TiO_2_, revealing the presence of Pt. Similarly, the weak peak at 44.3° proves the presence of Au in Au–TiO_2_. However, a weak peak is observed at 44.3° in the AuPt–TiO_2_ catalyst, which is speculated to be caused by the Au-coated Pt surface forming nanoparticles. The peaks of Au and Pt are consistent with the exposed faces of Au and Pt shown in Figure 1c. The UV–vis absorption spectra of obtained samples are shown in Figure 2b. Apparently, pure TiO_2_ only absorbs in the UV region. It is worth noting that the visible light absorption of the metal-modified composites is greatly enhanced. Au–TiO_2_ has a strong absorption peak around 548 nm due to the SPR effect of Au. Furthermore, a broad absorption band appears in the visible region of Pt NPs, which may be due to the intra-band transition (SPR absorption) and inter-band transition of Pt NPs, with a LSPR peak in the broad spectral range from near-UV to visible [33]. When the two noble metals are simultaneously reduced, the characteristic peak corresponding to the SPR effect of Au NPs disappears, presumably due to the interplay of the AuPt composites. As shown in Figure S4, the band gaps of above catalysts are calculated by Tauc plots, showing the influence of noble metals. Compared with TiO_2_ MSs, the band gaps of AuPt–TiO_2_, Pt–TiO_2_ and Au–TiO_2_ are narrowed from 3.22 eV. Pt–TiO_2_ has a narrower band gap (3.04 eV) than Au–TiO_2_, and AuPt–TiO_2_ shows the narrowest band gap of 3.01 eV due to the synergy of Au and Pt. In summary, the strong SPR effect of Au greatly improves the visible light absorption ability of AuPt–TiO_2_, while the Pt narrows the band gap [34,35]. The synergistic effect of AuPt causes the catalyst to have a better all-light absorption ability.

In order to determine the surface composition and chemical state, the X-ray photoelectron spectroscopy (XPS) spectra are obtained for TiO_2_, AuPt–TiO_2_, Pt–TiO_2_ and Au–TiO_2_. As shown in Figure 2c, the spectra of Ti 2p for pure TiO_2_ show three peaks at 458.78 eV, 464.48 eV and 472.08 eV, representing Ti 2p_3/2_, Ti 2p_1/2_ and Ti Sat. Correspondingly, there are three peaks representing Ti 2p_3/2_, Ti 2p_1/2_ and Ti Sat. at 458.78 eV, 464.38 eV and 471.98 eV in the spectra of Ti 2p for AuPt–TiO_2_. Compared to the spectra of Ti 2p for TiO_2_ MSs, the peaks in the spectra for AuPt–TiO_2_ are negatively shifted by 0.1 eV, due to the presence of oxygen vacancy. The peaks in the Ti 2p spectra of Pt–TiO_2_ and Au–TiO_2_ (Figure S5a) are also negatively shifted due to the existence of O_V_s. Three peaks at 529.98 eV, 531.98 eV and 533.48 eV can be observed in the O 1s spectra (Figure 2d), which are ascribed to the lattice oxygen (O_L_), the oxygen vacancy (O_V_) and the surface-absorbed oxygen (O_A_). The peaks area of O_v_ in the O 1s spectra for AuPt–TiO_2_ markedly increase due to the photo-deposition process, and similar phenomena also occur in the O 1s spectra for Pt–TiO_2_ and Au–TiO_2_ (Figure S5b). The Pt 4f and Au 4f spectra for AuPt–TiO_2_ (Figure 2e,f) exhibit valence distributions consistent with the single-metal-supported TiO_2_.

**Figure 3:**
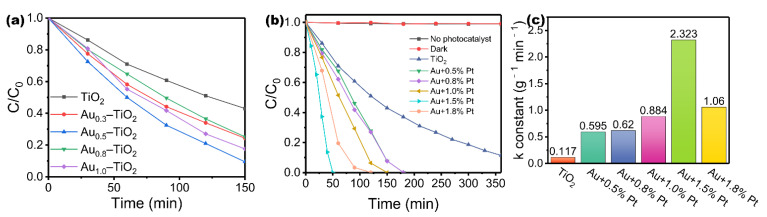
Under (UV–vis) light irradiation, (**a**) ethylene photodegradation curves of Au–TiO_2_ with different Au ratios, (**b**) ethylene photodegradation curves and (**c**) reaction rate constants of AuPt–TiO_2_ with different Pt ratios.

**Figure 4:**
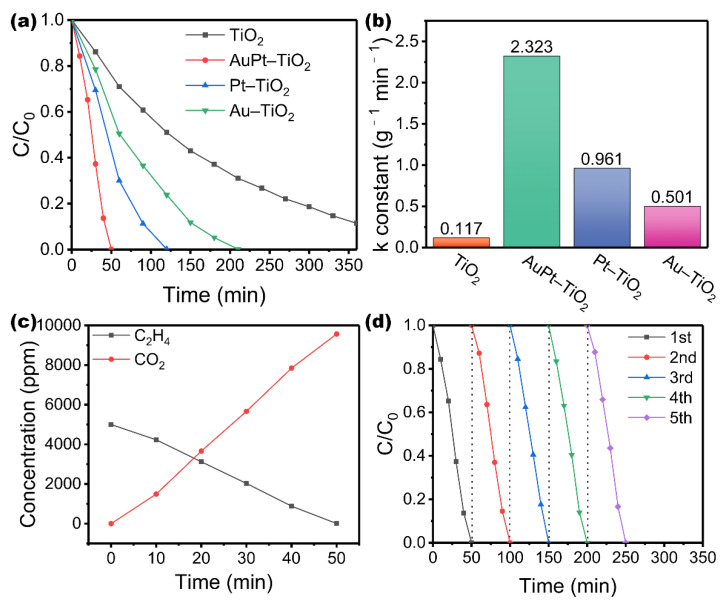
Under UV–vis light irradiation, (**a**) ethylene photodegradation curves and (**b**) reaction rate constants of pure TiO_2_, AuPt–TiO_2_, Pt–TiO_2_ and Au–TiO_2_ samples, (**c**) concentration curves of ethylene and carbon dioxide during photodegradation for AuPt–TiO_2_ and (**d**) five consecutive cycles’ stability of photocatalytic activity of AuPt–TiO_2_ for ethylene degradation.

The photodegradation activity of the above-mentioned catalysts is tested by photocatalytic ethylene degradation experiments. As shown in Figure 3, the ethylene concentration remains basically unchanged whether in the dark with a catalyst or in the light without a catalyst, indicating that the degradation of ethylene is a photocatalytic reaction. Figure 3a shows the photodegradation curves of TiO_2_ samples loaded with various mass ratios of Au. After loading Au NPs, the degradation rate is accelerated. It can be concluded that the SPR effect strengthens the visible light absorption of the catalyst, thereby enhancing the activity of photodegradation of ethylene. Meanwhile, loading different amounts of Au NPs has different effects on the improvement of TiO_2_ activity. When the loading amount of Au is 0.5 wt%, the activity of the catalyst is the best. Furthermore, we investigate the photodegradation performance of TiO_2_ loaded with 0.5 wt% Au and various amounts of Pt to explore the role of Pt. As shown in Figure 3b, the photodegradation activities of TiO_2_ supported with 0.5% Au and different amounts of Pt are further compared, which show TiO_2_ loaded with 0.5% Au and 1.5% Pt has the best ethylene degradation performance. Figure 3c shows the corresponding reaction rate constants for the catalysts. Clearly, TiO_2_ MSs degrade ethylene poorly with a rate constant of 0.117 g^–1^ min^–1^, and the reaction rate of the catalysts with the best loading ratio is about 20 times that of the pure sample. In order to prove the superiority of bimetallic loading on ethylene degradation, comparative TiO_2_ with 2.0% Au doping and 2.0% Pt doping is prepared. It can be observed from Figure 4a,b that the activity of AuPt–TiO_2_ is greater than that of Au–TiO_2_ and Pt–TiO_2_, and the reaction rate of AuPt–TiO_2_ is about 4.64 times that of Au–TiO_2_ and 2.42 times that of Pt–TiO_2_. In conclusion, utilizing a suitable bimetallic loading is very important in enhancing the performance of the catalyst. It also indicates that the synergistic effect of AuPt plays a major part in enhancing the activity of the catalyst. In order to study the ethylene mineralization ability of AuPt–TiO_2_, the relative concentrations of C_2_H_4_ and CO_2_ are calibrated to 5000 ppm and 0 ppm, respectively, before starting illumination (Figure 4c). After illumination, the ethylene content decreases gradually and is basically oxidized after 50 min. At the same time, the product CO_2_ is generated, and the final concentration is about 9600 ppm, which is close to the theoretical concentration. This result shows that most of the ethylene oxidation is generated into CO_2_. The mineralization rate of ethylene is approximately 96%, which indicates that AuPt–TiO_2_ possesses ideal and complete ethylene photodegradation ability.

Under the same experimental conditions, the stability of the photocatalytic degradation of ethylene is investigated by five cyclic experiments. As shown in Figure 4d, the AuPt–TiO_2_ photocatalyst maintains its initial excellent stability after five consecutive cyclic photodegradation experiments, indicating that the AuPt–TiO_2_ photocatalyst possesses excellent recyclability. TEM images of AuPt–TiO_2_ after five consecutive cyclic photodegradation experiments (Figure S6) show no significant changes in structure. Similarly, the peaks in the XRD patterns of AuPt–TiO_2_ are consistent before and after cyclic photodegradation experiments (Figure S7). The Ti 2p and O 1s spectra for AuPt–TiO_2_ show no significant peak shift, and the spectra of Au 4f and Pt 4f also maintain the corresponding valence distribution (Figure S8). As a photocatalyst for ethylene degradation, AuPt–TiO_2_ has the advantages of high efficiency, long-term stability and strong mineralization ability, indicating an ideal application prospect.

**Figure 5:**
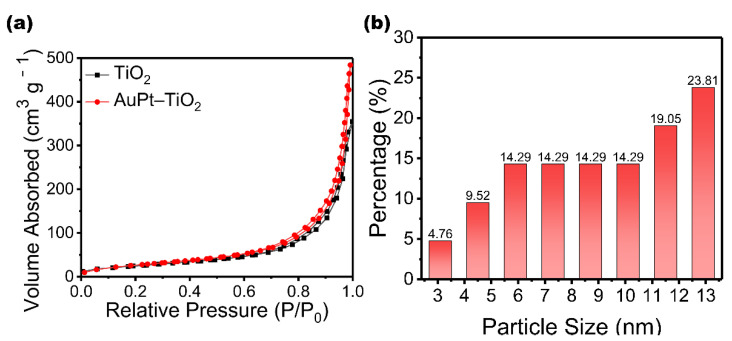
(**a**) N_2_ adsorption–desorption isotherms and (**b**) particle size distribution of TiO_2_ MSs and AuPt–TiO_2_.

**Figure 6:**
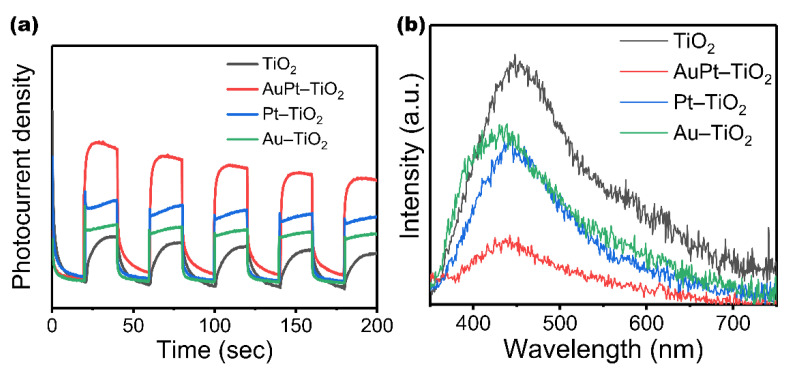
(**a**) Transient photocurrent spectra and (**b**) PL spectra of TiO_2_, AuPt–TiO_2_, Pt–TiO_2_ and Au–TiO_2_.

To explore the reasons for the enhanced photodegradation ethylene ability of AuPt–TiO_2_, a series of tests are conducted. Surface area and porosity of TiO_2_ MSs and AuPt–TiO_2_ are illustrated in Table S1, showing that AuPt–TiO_2_ has a larger surface of 104.05 m^2^ g^−1^ than that of TiO_2_ MSs, 92.05 m^2^ g^−1^. Similarly, TiO_2_ MSs also have smaller pore volume and pore size than AuPt–TiO_2_. As shown in Figure 5a, TiO_2_ MSs own a strong adsorption capacity for nitrogen, which reflects the large specific surface area brought by the nanoflower morphology. In addition, nanoparticles are attached to ultrathin TiO_2_ nanosheets, which further increase the specific surface area of AuPt–TiO_2_. This endows AuPt–TiO_2_ with a strong gas adsorption capacity, which improves the transfer rate of interfacial charges and enhances the separation ability of photogenerated electrons and holes. Figure 5b shows the size distribution of nanoparticles on AuPt–TiO_2_ obtained from TEM imagery (Figure 1b); the size of the nanoparticles varies from 3.1 nm to 12.9 nm. In this paper, the BET surfaces show an opposite trend to previous reports, and we speculate as to the reason for this phenomenon [36,37,38]. The AuPt distributed in the pores increase the BET surface and pore volume due to their radius, which is smaller than the pore size. Additionally, the AuPt particles themselves have a certain surface area and are deposited on the TiO_2_ surface [39,40,41]. In addition, the H^+^ produced a certain acid etching effect during the photo-reduction process, increasing the pore size of AuPt–TiO_2_. In addition, the separation ability of photogenerated electrons and holes in the catalyst is further investigated by photocurrent intensity tests and PL spectroscopy. Figure 6a shows the transient photocurrent response results of TiO_2_, AuPt–TiO_2_, Pt–TiO_2_ and Au–TiO_2_. All tested samples show fast and uniform photocurrent intensity. The photocurrent response of pure TiO_2_ is poor, but the photocurrent response of Pt–TiO_2_ and Au–TiO_2_ after single-metal loading is stronger than that of pure TiO_2_ because Pt and Au are more favorable for capturing electrons. Notably, the photocurrent response of AuPt–TiO_2_ is further improved in the bimetallic system. Figure 6b shows the PL intensities of AuPt–TiO_2_, Pt–TiO_2_ and Au–TiO_2_. The lower the PL intensity, the stronger the separation ability of photogenerated electron–hole pairs. It can be seen that the order of intensity was AuPt–TiO_2_ < Pt–TiO_2_ < Au–TiO_2_ < pure TiO_2_. Among them, the intensity of AuPt–TiO_2_ is the lowest, which indicates that AuPt–TiO_2_ has the strongest electron–hole separation ability, consistent with the results of the photocurrent test.

**Figure 7:**
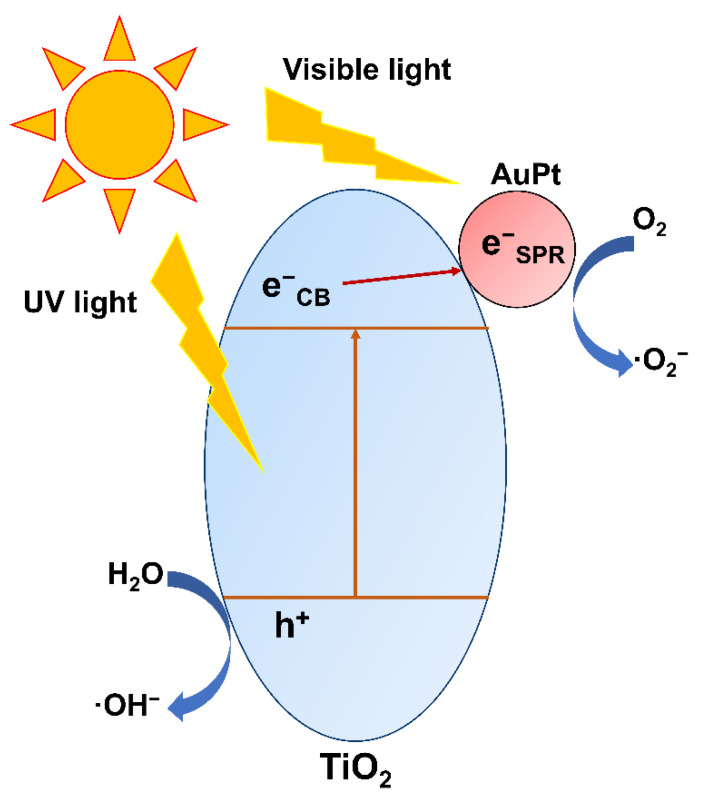
The possible reaction mechanism for photocatalytic degradation of ethylene.

These results are in accordance with the photodegradation activity, and also indicate that the synergistic effect of Au–Pt bimetals can greatly improve the separation efficiency of photogenerated electrons and holes, resulting in higher ethylene photodegradation activity of AuPt–TiO_2_. For the above, we propose the following mechanisms for the ethylene photodegradation ability of AuPt–TiO_2_: On the one hand, the SPR absorption provided by Au enhances the visible light absorption of AuPt–TiO_2_. On the other hand, although Pt possesses only a weak SPR absorption, it can significantly improve the light absorption capacity and carrier separation efficiency of AuPt–TiO_2_, which is proven by the transient photocurrent spectra and PL spectra. In summary, the synergistic effect of Au and Pt enhances the visible light absorption and the carrier separation efficiency of AuPt–TiO_2_, resulting in a significantly enhanced photodegradation ability. Herein, we propose a feasible mechanism for the photocatalytic ethylene degradation as shown in Figure 7. Because of the wide band gap of 3.2 eV, anatase TiO_2_ shows only absorption of UV light, which cannot be excited by visible light. Due to the SPR resonance, Au and Pt show excellent absorption in the visible light area. Hence, not only TiO_2_ can process the single-electron reduction of oxygen to ·O_2_^−^, but the SPR-induced electrons are also produced on the surface of AuPt. Furthermore, AuPt, as an electronic capture center, receives electrons from the conduction band of TiO_2_ and prolongs the life of electrons. The synergy of e^−^_CB_ and e^−^_SPR_ enhances the photocatalytic degradation ability of AuPt–TiO_2_ under UV–vis light. In addition, the photogenerated holes help the convert from H_2_O to ·OH^−^, which enhances the mineralization of ethylene under the synergistic effect of ·O_2_^−^.

## 4. Conclusions

In this work, Au and Pt-co-loaded TiO_2_ at the suitable Au/Pt ratio is an ideal catalyst for photodegradation of ethylene. AuPt–TiO_2_ shows relatively excellent ethylene photodegradation ability, which is 19.9, 4.64 and 2.42 times that of TiO_2_ MSs, Au–TiO_2_ and Pt–TiO_2_. The results show that the ethylene photodegradation ability benefits from the synergy of Au and Pt. This work may help to deepen the research of enhancing the non-polar small-molecule degradation ability of semiconductors.

## Data Availability

The data presented in this study are available on request from the corresponding author.

## Supplementary Materials

The following supporting information can be downloaded at: https://www.mdpi.com/article/10.3390/nano12183221/s1, Figure S1: (a,b) TEM, (c) HRTEM images of TiO_2_ MSs; Figure S2: SEM images of (a) Au-TiO_2_ and (b) Pt-TiO_2_; Figure S3: (a) EDS spectrum and (b,c) EDS line scan spectra of AuPt-TiO_2_; Figure S4: Tauc plots of TiO_2_ MSs, AuPt- TiO_2_, Pt-TiO_2_ and Au-TiO_2_; Figure S5: (a) Ti 2p and (b) O 1s XPS spectra of TiO_2_, Pt- TiO_2_ and Au-TiO_2_; Figure S6: (a,b)TEM, (c) HRTEM images of AuPt-TiO_2_ after 5 cycels stability test; Figure S7: XRD patten of AuPt-TiO_2_ before and after 5 cycels stability test; Figure S8: (a) Ti 2p, (b) O 1s, (c) Au 4f and (d) Pt 4f XPS spectra of AuPt-TiO_2_ before and after 5 cycels stability test; Table S1: Surface area and porosity of TiO_2_ MSs and AuPt-TiO_2_.
