# Supplementary material for: Synergy of Au–Pt for Enhancing Ethylene Photodegradation Performance of Flower-like TiO2
Source: Nanomaterials (Basel). 2022 Sep 16;12(18):3221. doi: 10.3390/nano12183221 (PMC9505558; doi:10.3390/nano12183221)
Supplement: Supplementary file 1 [file nanomaterials-12-03221-s001.zip › nanomaterials-1843852-supplementary.pdf]

nanomaterials

Synergy of Au–Pt for Enhancing Ethylene Photodegradation Performance of Flower-like TiO_2

Wanzhen Meng [1], Yunrui Zhao [1], Dujuan Dai [1], Qianqian Zhang [1,*], Zeyan Wang [1], Yuanyuan Liu [1], Zhaoke Zheng [1], Hefeng Cheng [1], Ying Dai [2], Baibiao Huang [1] and Peng Wang [1,*]

[1] State Key Laboratory of Crystal Materials, Shandong University, Jinan 250100, China
[2] School of Physics, Shandong University, Jinan 250100, China
* Correspondence: zhangqianqian@sdu.edu.cn (Q.Z.); pengwangicm@sdu.edu.cn (P.W.)

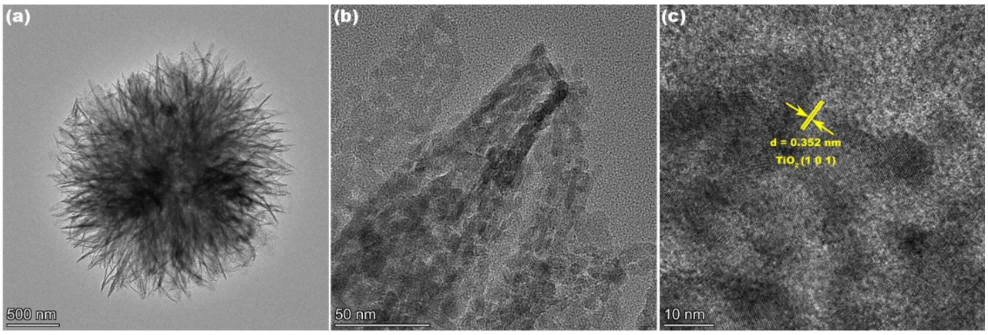

Figure S1. a, b)TEM, c) HRTEM images of TiO_2 MSs.

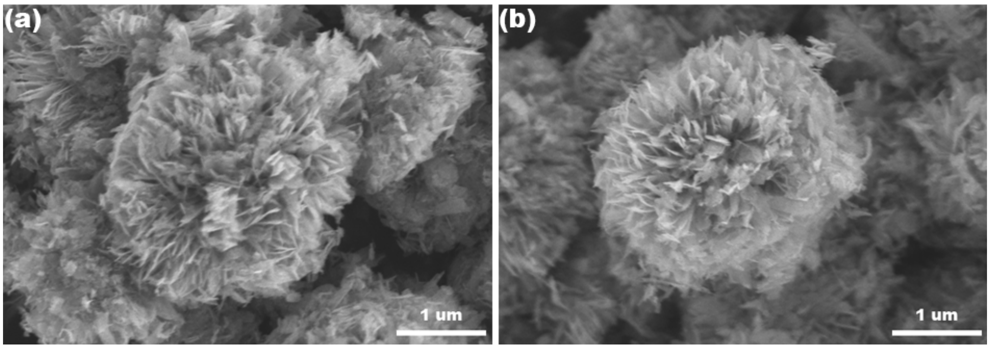

Figure S2. SEM images of a) Au-TiO_2 and b) Pt-TiO_2.

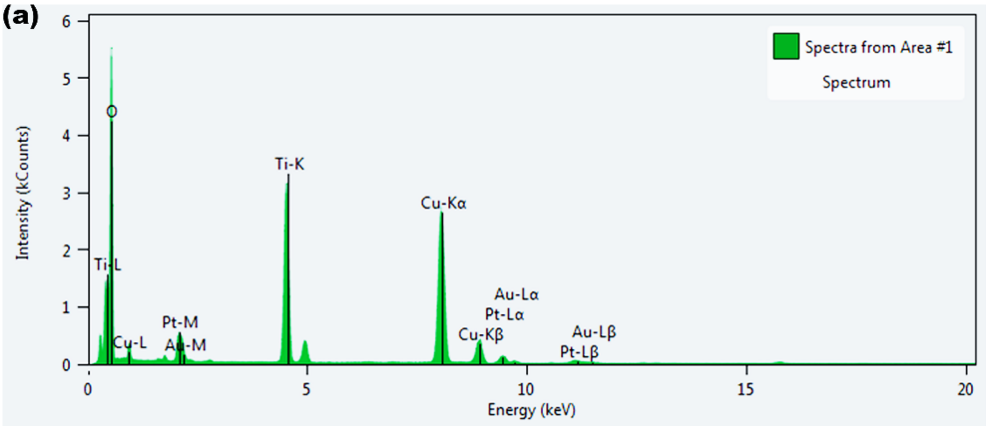

Z	Element	Family	Atomic Fraction (%)	Atomic Error (%)	Mass Fraction (%)	Mass Error (%)	Fit error (%)
8	O	K	68.13	7.23	38.13	2.67	1.60
22	Ti	K	30.22	5.11	50.59	7.54	0.14
78	Pt	L	1.28	0.19	8.73	1.12	0.23
79	Au	L	0.37	0.06	2.55	0.33	0.66

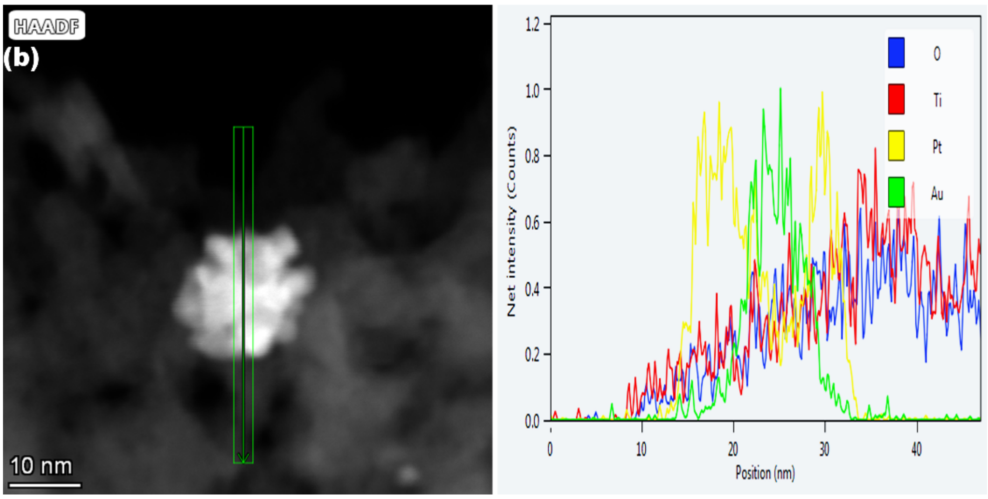

Figure S3. a) EDS spectrum and b) EDS line scan spectra of AuPt-TiO_2.

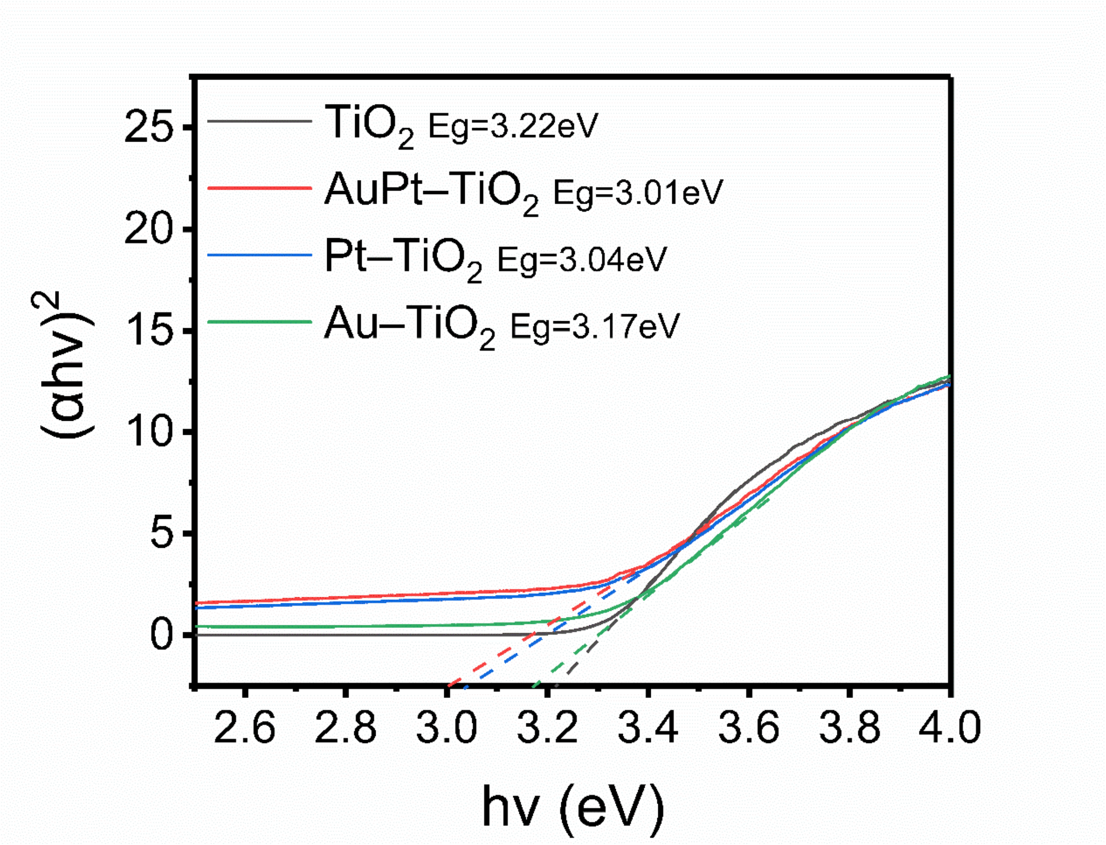

Figure S4 Tauc plots of TiO_2 MSs, AuPt- TiO_2, Pt-TiO_2 and Au-TiO_2.

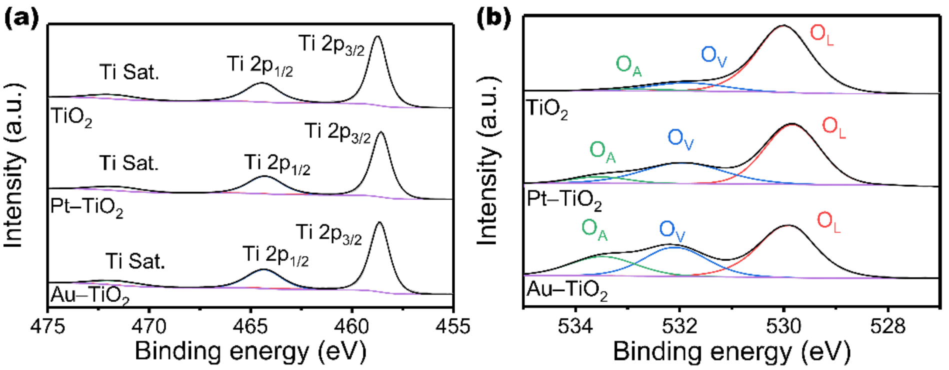

Figure S5. a) Ti 2p and b) O 1s XPS spectra of TiO_2, Pt- TiO_2 and Au-TiO_2.

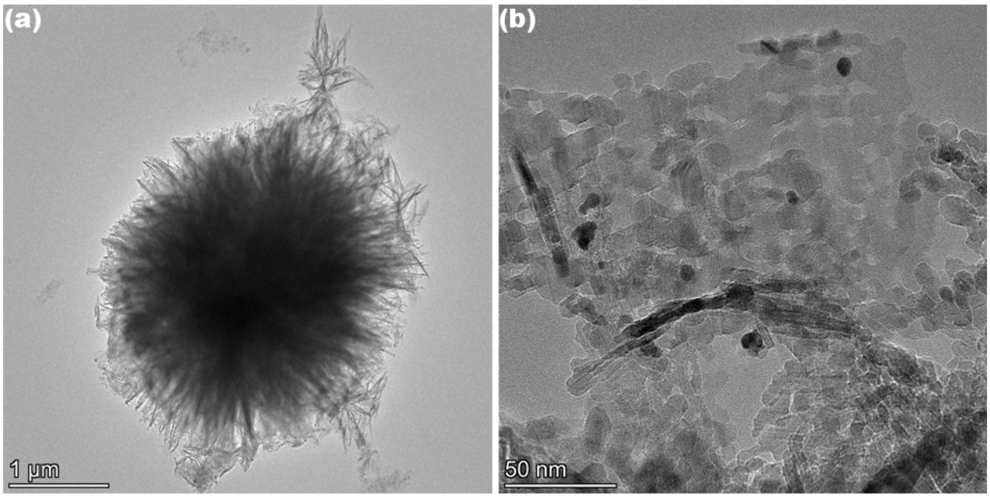

Figure S6. a, b)TEM, HRTEM images of AuPt-TiO_2 after 5 cycels stability test.

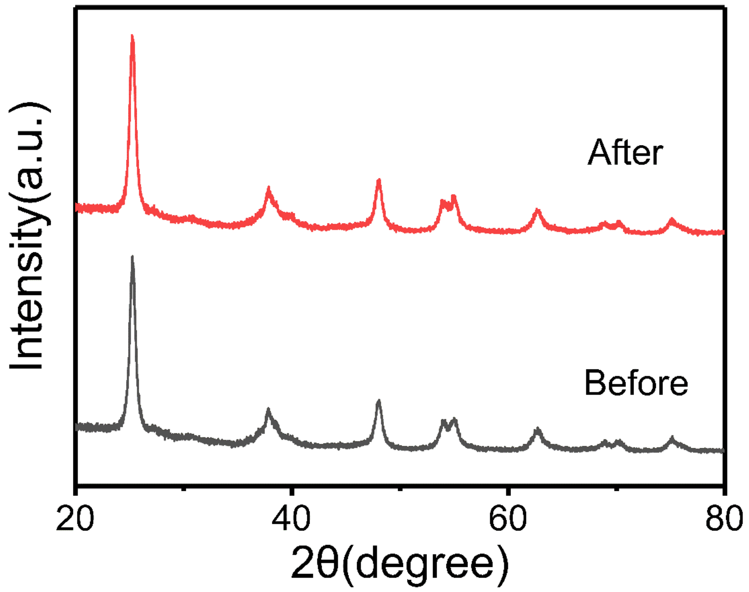

Figure S7. XRD patten of AuPt-TiO_2 before and after 5 cycels stability test.

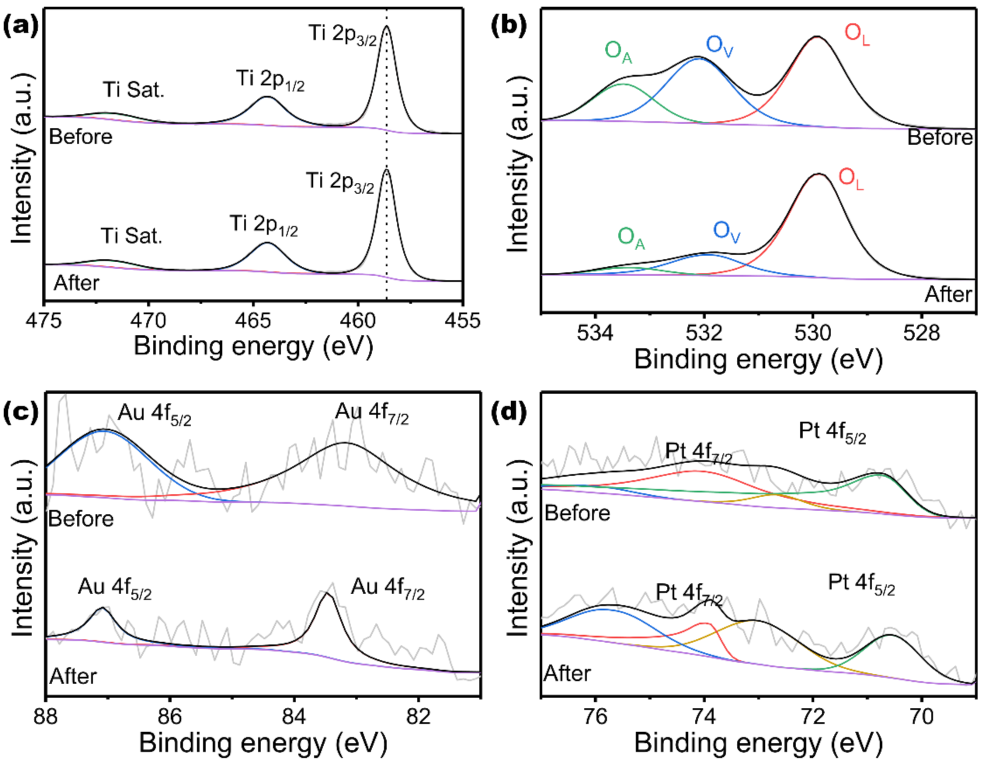

Figure S8. a) Ti 2p, b) O 1s, c) Au 4f and d) Pt 4f XPS spectra of AuPt-TiO_2 before and after 5 cycels stability test.

Table S1. Surface area and porosity of TiO_2 MSs and AuPt-TiO_2.

Samples	Surface Area (m^2/g)	Pore Volume (cm^3/g)	Pore Size (nm)
TiO_2 MSs	92.05	0.5488	11.9
AuPt-TiO_2	104.5	0.7487	14.3
